# EFFECTS OF ADVANCE CARE PLANNING TRAINING ON STUDENTS’ KNOWLEDGE, COMFORT, AND CONFIDENCE

**DOI:** 10.1093/geroni/igae098.3108

**Published:** 2024-12-31

**Authors:** Stacy Barnes, Susan Breakwell

**Affiliations:** Marquette University College of Nursing, Milwaukee, Wisconsin, United States; Marquette University, Milwaukee, Wisconsin, United States

## Abstract

Students report feeling unprepared to engage in advance care planning (ACP).

**Methods:**

A 2-hour, interprofessional workshop was held in-person in April 2018 and 2019, and virtually in 2021 and 2022 for students from medicine, nursing, physician assistant, speech pathology, counseling psychology, biomedical sciences, communication, and sociology (N=223). The workshop aimed to educate students about ACP, foster exploration of personal preferences for end-of-life care, and empower them to initiate ACP conversations with others. It incorporated highly engaging activities including personal reflection, drawing, writing, video clips, case presentations, as well as small and large group discussions. A Qualtrics survey, using a retrospective post- then pre- questionnaire design, elicited feedback 1-day post-workshop.

**Results:**

Regardless of delivery format, respondents rated the workshop highly (97% excellent or good) and liked interacting with peers from other disciplines in small group discussions best. Paired-samples t-tests revealed statistically significant differences following the workshop in four of five areas (p< 0.001) i.e., increased understanding of advance directives, comfort completing their own advance directive, comfort talking with their family and/or personal doctor, and confidence in starting an ACP conversation with others. No significant change was detected in students’ comfort exchanging ACP ideas in a team discussion (p=0.19).

**Discussion:**

A workshop can increase students’ self-reported knowledge, comfort, and confidence with ACP, as applied to themselves and/or family. However, results suggest working in an interprofessional healthcare team requires additional training and experience. These findings can help guide ACP curricula in health professions education.

